# Identification of a Desaturase Involved in Mycolic Acid Biosynthesis in *Mycobacterium smegmatis*

**DOI:** 10.1371/journal.pone.0164253

**Published:** 2016-10-14

**Authors:** Albel Singh, Cristian Varela, Kiranmai Bhatt, Natacha Veerapen, Oona Y. C. Lee, Houdini H. T. Wu, Gurdyal S. Besra, David E. Minnikin, Nagatoshi Fujiwara, Kanae Teramoto, Apoorva Bhatt

**Affiliations:** 1 School of Biosciences and Institute of Microbiology and Infection, University of Birmingham, Edgbaston, Birmingham, B15 2TT, United Kingdom; 2 Heart of England NHS Trust, Birmingham, United Kingdom; 3 Department of Food and Nutrition, Faculty of Contemporary Human Life Science, Tezukayama University, Nara City, Nara, 631-8585, Japan; 4 Advanced and Fundamental Technology Center, JEOL Ltd., Akishima City, Tokyo, 196–8558, Japan; Centre National de la Recherche Scientifique, FRANCE

## Abstract

Mycolic acids are unique long chain fatty acids found in the cell walls of mycobacteria including the tubercle bacillus, *Mycobacterium tuberculosis*. The introduction of double bonds in mycolic acids remains poorly understood, however, genes encoding two potential aerobic desaturases have been proposed to be involved in this process. Here we show that one of these genes, *desA1*, is essential for growth of the saprophytic *Mycobacterium smegmatis*. Depletion of *desA1* in a *M*. *smegmatis* conditional mutant led to reduction of mycolic acid biosynthesis and loss of viability. The DesA1-depleted cells exhibited two other phenotypes: using ^14^[C]-labelling, we detected the accumulation of minor mycolic acid-related species that migrated faster in a silver TLC plate. Spiral Time of Flight Mass Spectroscopic analysis suggested the presence of species with sizes corresponding to what were likely monoenoic derivatives of α-mycolic acids. Additionally, conditional depletion led to the presence of free fatty acyl species of lengths ~C_26_-C_48_ in the lysing cells. Cell viability could be rescued in the conditional mutant by *Mycobacterium tuberculosis desA1*, highlighting the potential of *desA1* as a new drug target in pathogenic mycobacteria.

## Introduction

**Fig 1:**
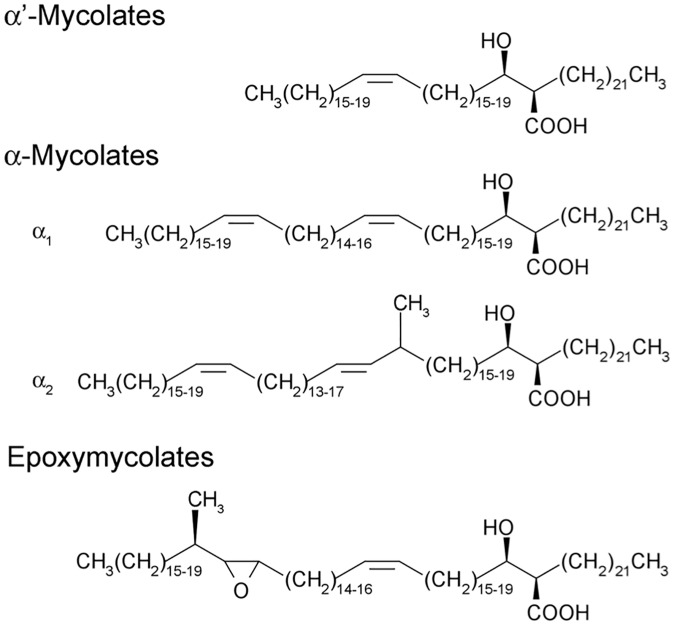
Structures of mycolic acid subclasses found in *Mycobacterium smegmatis*.

*Mycobacterium tuberculosis*, the bacterium that causes tuberculosis (TB), synthesises a distinct class of long-chain fatty acids termed mycolic acids. These α-alkyl, β-hydroxy fatty acids are abundant in the waxy cell wall of the tubercle bacillus and the cell walls of other mycobacteria including the leprosy bacillus *Mycobacterium leprae*, bovine TB-causing *Mycobacterium bovis* and the saprophytic *Mycobacterium smegmatis*. Mycolic acids are found either esterified to the terminal carbohydrate moieties of the peptidoglycan-arabinogalactan complex, or as part of the outer glycolipids, glucose monomycolate, trehalose monomycolate and trehalose dimycolate (TDM; also known as cord factor). A combination of early biochemical studies and subsequent development of mycobacterial genetics has helped unravel the pathways leading to the biosynthesis of mycolic acids [1–3]. Mycolic acids in mycobacteria consist of a longer mero-chain, and a shorter α-chain. Biosynthesis of mycolic acids is initiated by the ‘housekeeping’ fatty acid synthase, the multidomain fatty acyl synthase—I (FAS-I) that produces C_16_–C_18_ and C_24_–C_26_ fatty acids in a bimodal fashion. The latter forms the α-chain of mycolic acids. The mero-chain is synthesised by the multienzyme fatty acid synthase II (FAS-II) that extends FAS-I derived palmitoyl/stearoyl-S-CoA to yield fatty acids of chain length C_60_. The mero-chain then undergoes Claisen condensation with the α-chain; a reaction catalysed by a polyketide synthase, Pks13 to yield a 3-oxo, α-alkyl fatty acid [4,5], which is subsequently reduced by a mycolyl reductase to form a mycolic acid [6,7]. Core to mycolic acid structure is the generation of double bonds found in the mero-chain. *M*. *smegmatis* produces three subclasses of mycolic acids: α, α’ and epoxy mycolic acids (Fig 1); α mycolic acids contain two double bonds in the mero-chain, while α’ mycolates contain one double bond. In *M*. *tuberculosis*, formation of *cis*-double bonds can be a precursor for subsequent modification steps such as cyclopropanation [8–10]. Asselineau *et al*. [11] hypothesised that a C_24:0_ fatty acyl chain is first desaturated to a *cis*-Δ5-C_24:1_ intermediate which is then elongated by FAS-II, and subsequently desaturated to yield *cis*-Δ3, *cis*-Δ15-C_34:2_ merochains which are eventually elongated further and incorporated into mycolic acids. In *Escherichia coli*, fatty acid desaturation is brought about by the action of FabA, a dual function enzyme that catalyses both the formation of a *trans*-double bond in a fatty acyl-chain, and its subsequent isomerisation to the *cis* configuration [12]. In the absence of FabA homologues in the genomes of mycobacteria, two alternative pathways to double bond formation have been suggested [3]. In *Streptococcus pneumoniae*, the novel isomerase/dehydratase pair FabM/FabZ diverts fatty acid biosynthesis to the unsaturated mode [13]. The *M*. *tuberculosis* homologues *echA10* and *echA11*, have been proposed as candidates, though their predicted non-essentiality makes it unlikely that they play any role in mycolic acid desaturation [3]. An alternative pathway involving a Δ5 desaturase has also been proposed to convert C_24:0_ to C_24:1_ prior to further elongation to yield the α-mycolate mero-chain [11]. Such a desaturase would require molecular oxygen, and interestingly the *M*. *tuberculosis* H37Rv genome contains genes encoding three candidate aerobic desaturases: *desA1*, *desA2* and *desA3* [14]. Of these, *desA3* has been shown to be involved in the biosynthesis of oleic acid, rather than mycolic acids [15]. Homologues of *M*. *tuberculosis desA1*, *desA2* and *desA3* are found in other mycobacteria incuding *M*. *bovis*, *M*. *leprae* and *M*. *smegmatis*, though *desA3* is predicted to be a pseudogene in *M*. *leprae* [16,17]. Dyer *et al*. [18] attempted structural studies on DesA1 and DesA2, and while the authors were unable to obtain soluble DesA1, the solved structure of DesA2 [18] revealed that DesA2 exists as a homodimer and is structurally related to plant fatty acid desaturases. However, differences in the variable metal binding pockets of DesA2, including a possible alternative access of ligand to the metal center, suggested a structurally distinct substrate for this mycobacterial fatty acid desaturase. This, and the extent of disorder in the inter-subunit region of the protein indicated a specialised role for DesA2. Here, we have investigated the role of *desA1* in the desaturation of mycolic acid mero-chains in the fast growing *M*. *smegmatis*.

## Results

### DesA1 is essential for mycobacterial viability during growth in laboratory media

**Fig 2:**
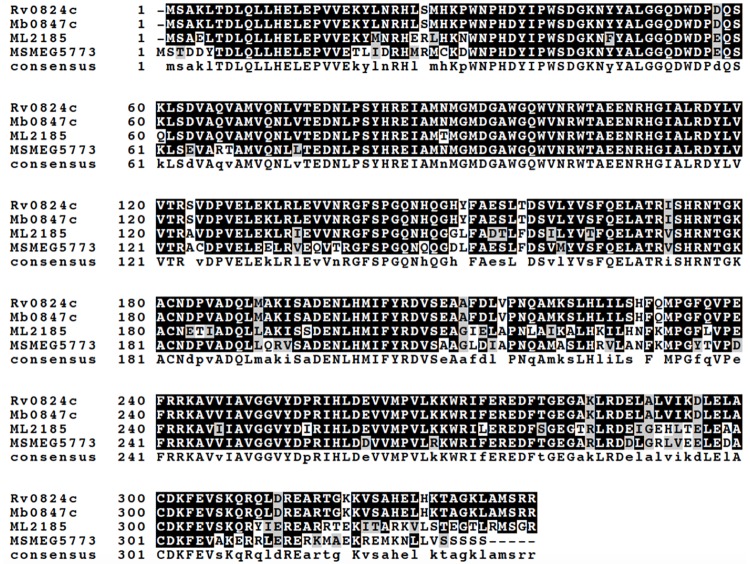
Multiple sequence alignment of *M*. *tuberculosis* DesA1 (Rv0824c) and homologues from *M*. *bovis* (Mb0847c), *M*. *leprae* (ML2185) and *M*. *smegmatis* (MSMEG5773). Consensus residues found in all four amino acid sequences are indicated by uppercase letters.

**Fig 3:**
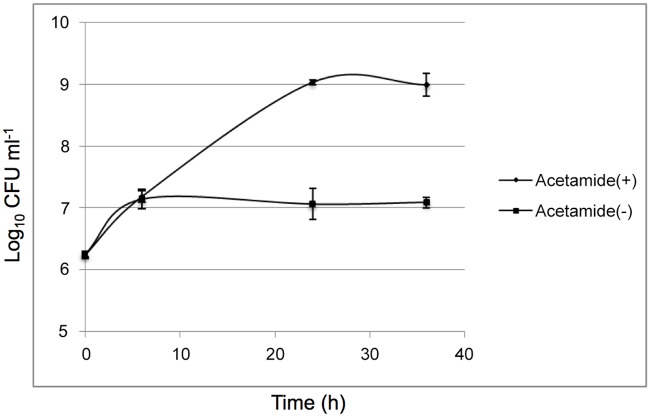
Conditional depletion of DesA1 leads to lysis of the Δ*MsdesA1* mutant. Cultures were grown for up to 36h in TSB in the presence (+Acetamide) or absence (-Acetamide) of inducer. Loss of viability was more distinct on solid media (See control strain in Fig 12).

The protein encoded by *M*. *tuberculosis desA1* (Rv0824c) shares 80% identity and 90% similarity with the *M*. *smegmatis* homologue encoded by *MSMEG5773* (multiple sequence alignment shown in Fig 2). To test the potential essentiality of *desA1*, we first attempted to generate a null mutant of *desA1* in *M*. *smegmatis* by Specialized Transduction [19,20] using phΔMSMEG5773, a recombinant phage designed to replace *M*. *smegmatis desA1* (*MSMEG5773*) with a hygromycin resistance cassette (*hyg*). Repeated transductions of *M*. *smegmatis* mc^2^155 with phΔ*MSMEG5773* failed to generate any hygromycin resistant transductants suggesting that the gene was likely to be essential for growth in laboratory media. We then proceeded to demonstrate the essentiality of *desA1* using CESTET, a gene essentiality-testing tool for *M*. *smegmatis* [21]. First, we generated a merodiploid strain containing a second, integrated copy of *desA1* under the control of an acetamide-inducible promoter. Following transduction of the merodiploid strain, we were able to obtain hygromycin resistant colonies on agar plates containing acetamide. One such transductant was confirmed by Southern blot to have the native copy of *desA1* replaced with *hyg*, and was designated Δ*MsdesA1*. When broth cultures of Δ*MsdesA1* were transferred to fresh medium containing acetamide, they continued growing, however when cultures were transferred to medium without acetamide, a relative decrease in viable counts was observed (Fig 3) indicating that *desA1* was essential for growth of *M*. *smegmatis* in laboratory media. All together, these results validated the essentiality of *desA1* in *M*. *smegmatis*.

### Loss of *M*. *smegmatis* DesA1 leads to loss of mycolic acid biosynthesis

**Fig 4:**
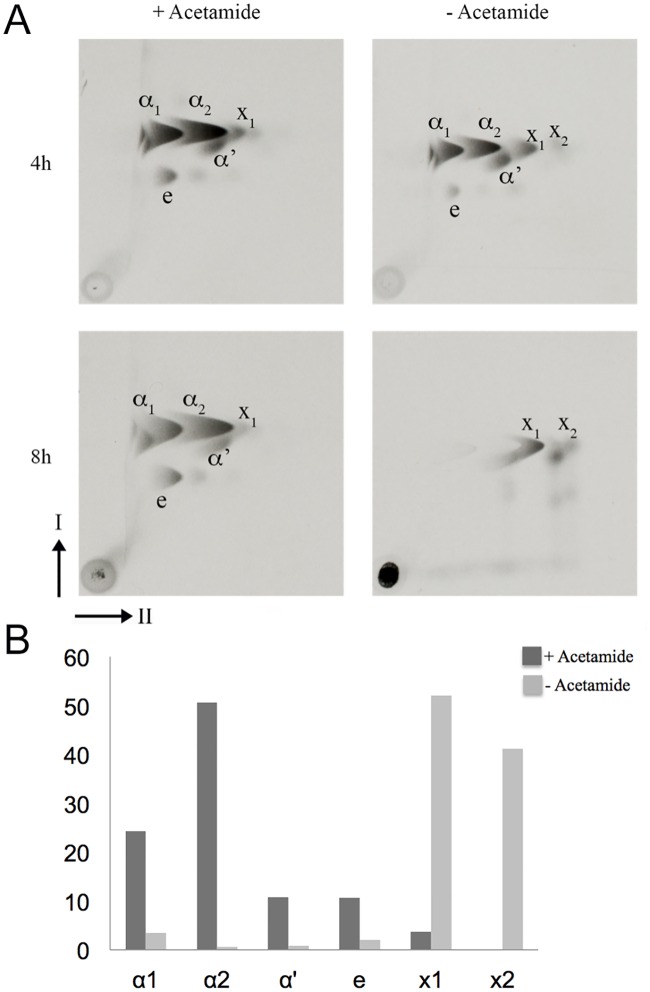
Argentation TLC of ^14^C-labelled methyl esters of cell wall mycolic acids (MAMES) extracted from the Δ*MsdesA1* mutant grown in the presence or absence of acetamide. (A) Direction-I separated in petroleum ether:acetone (95:5, v/v); direction-II separated thrice in AgNO_3_ treated silica gel in petroleum ether:ethyl acetate (9:1, v/v). The cyclopropanated derivative is indicated as X_1_ and the faster-migrating species that co-migrated with α-MAMES in direction-I are indicated as X_2_. The clear ‘imprint’ appearing superimposed on the comet shaped X_2_ spot in the 8h –acetamide TLC is likely due to unlabelled α_2_ mycolates synthesised at the early stages of DesA1 depletion. (B) Bar graph showing the relative amounts of each ^14^C-labelled mycolate subspecies indicated as a percentages of the total amounts of ^14^C-labelled mycolic acids detected on the TLC plates, as determined by densitometry.

To determine the effect of loss of DesA1 on mycolic acids we extracted ^14^C-labelled mycolic acids methyl esters (MAMEs) from cultures of the Δ*MsdesA1* strain grown in the presence or absence of acetamide, and analysed the extracts by 2D-argentation TLC. The second TLC dimension involves separation of saturated and unsaturated species in an AgNO_3_-treated section of the TLC plate, as the presence of double bonds retards migration in this dimension. Cultures grown in the absence of acetamide showed a decrease in the biosynthesis of subclasses of α, α´ and epoxy mycolic acids over time (Fig 4). Additionally, we also observed a previously described cyclopropanated α-mycolate species [22] in both cultures (Labelled X_1_ in Fig 4A). Levels of X_1_ did not decrease following depletion of DesA1, even at the later time point. This was surprising, as cyclopropane ring formation requires double bond formation.

In addition to the above phenotypes, DesA1-depleted cultures also revealed the presence of faint but detectable levels of extra species of fatty acyl methyl esters that predominantly co-migrated with α-MAMEs in the first dimension, but migrated further than the α-MAMEs in the second, AgNO_3_-containing dimension in the TLC plate (labelled ‘X_2_’ in Fig 4A). This result suggested that the faster migrating spots (X_2_) in the 2D-TLC plates were likely fatty acyl methyl esters related to α-mycolic acids.

To further characterise mycolic acid species in the conditional mutant, we first subjected purified, non-labelled α-MAMES to high-resolution matrix-assisted laser desorption /ionization time-of-flight mass spectrometry. Unlike ^14^C-labelled cultures, using non-labelled cells does not offer an opportunity to study mycolates in a time dependent fashion. Instead, MAMEs extracted from non-labelled, no-acetamide cultures represent both ‘regular’ mycolates produced during the early stages of DesA1 depletion, and those produced at later stages of DesA1 depletion. The levels of these early stage ‘regular’ mycolates (which would also be present in the +acetamide cultures) would thus dominate in any subsequent analysis. Thus, the unkown, accumulating MAMEs (species X_2_) in the DesA1-depleted cultures would represent only a fraction of the total MAMEs extracted from non-labelled cell pellets.

**Fig 5:**
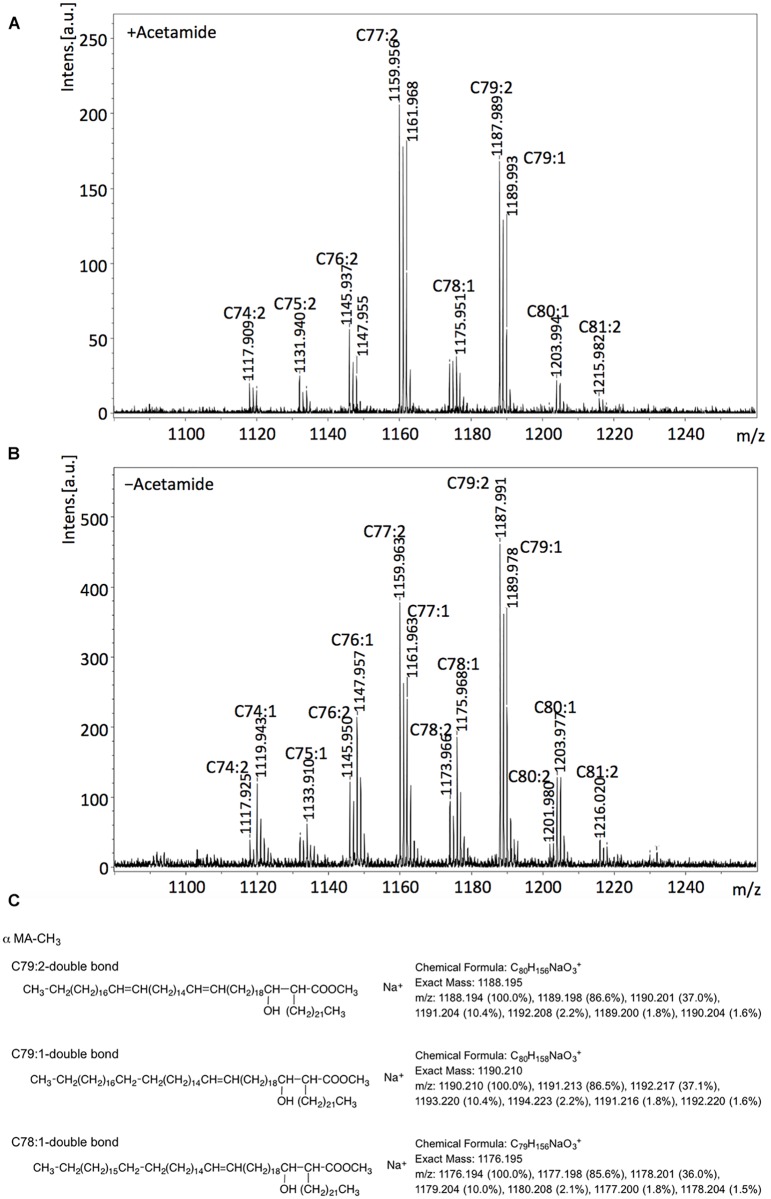
MALDI Mass Spectroscopy analysis of α-MAMES purified from the Δ*MsdesA1* mutant grown in the presence or absence of acetamide. A, mass spectra of α-MAMES extracted from (A) + acetamide cultures and (B)–acetamide cultures. (C) Example of predicted sizes and structures based on observed masses.

The molecular masses calculated for the observed MAME species in cultures grown the presence of acetamide indicated that dienoic C_77:2_ and C_79:2_ were the major α-MAMEs in the non-depleted cultures (+acetamide; Fig 5A) though other species, including, surprisingly those with masses corresponding to monoenoic derivatives, were also present. While the DesA1 depleted cultures (-acetamide; Fig 5B) showed the presence of same species, we detected slightly higher relative peak intensities for MAMEs with sizes predicted to represent monoenoic derivatives C_74:1_, C_76:1_,C_78:1_ and C_80:1_ (Fig 5C).

**Fig 6:**
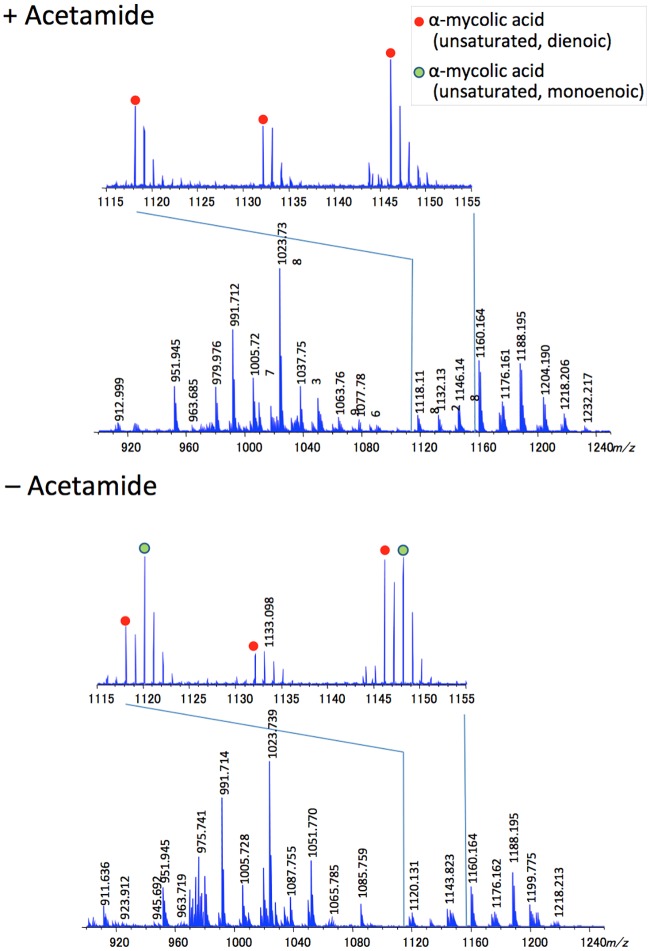
MALDI Spiral-TOF Mass Spectroscopy analysis of total MAMES purified from the Δ*MsdesA1* mutant grown in the presence or absence of acetamide.

We further analysed total mycolates extracted from cultures of Δ*MsdesA1* grown in the presence or absence of acetamide, using MALDI-TOF MS with a spiral trajectory [23,24]. We chose this method over conventional MALDI-TOS MS as it gives high mass resolution and accuracy, and enables the separation of adjacent peaks with a 0.036 Da mass difference. Consistent with our findings with purified α-MAMES, we observed molecular masses corresponding to both monoenoic and dienoic α-MAMES (Fig 6; S1 and S2 Figs, S1 and S2 Tables). Given the accuracy of the method in distinguishing between species differing by 0.036 Da mass differences, we were also able to account for epoxy mycolates and distinguish these from potentially monoenoic α-mycolates of similar masses. For example the epoxy mycolate species C_78_H_152_O_4_Na (*m/z* 1176.158) could be differentiated from monoenoic α-mycolate C_79_H_156_O_3_Na, (*m/z* 1176.194). However, we could not distinguish between dienoic α-mycolates and their cyclopropanated derivatives as species with the same number of carbons would have an identical mass.

While ^14^C-acetate labelling afforded a sensitive method for visualising newly synthesised mycolates extracted from smaller culture volumes within defined time frames, further MS and NMR analysis required non-radioactive, unlabelled samples derived from scaled up cultures. Bacterial lysis limited the amount of cell material we could collect for this analysis. Furthermore, as mentioned earlier, unlabelled cell pellets would yield mycolates synthesised both in the early and late stages of depletion and thus the observed differences between the relative levels of monenoic and dienoic mycolates were perhaps under-represented here. Given the relatively low levels of species X_2_ expected in non-labelled samples, we were unable to further study structural features of this species from DesA1-depleted cultures.

In summary, the 2D-argentation TLC results and subsequent MS analysis indicate that loss of DesA1 leads to changes in the relative proportions of monoenoic and dienoic derivatives of α-MAMEs following loss of DesA1 function. We cannot conclude that depletion of DesA1 led to an overall accumulation of monoenoic derivatives of α-mycolic acids. However, using Mass Spectroscopy, we were able to show changes in the relative proportions of monoenoic and dienoic derivatives following loss of DesA1 function.

**Fig 7:**
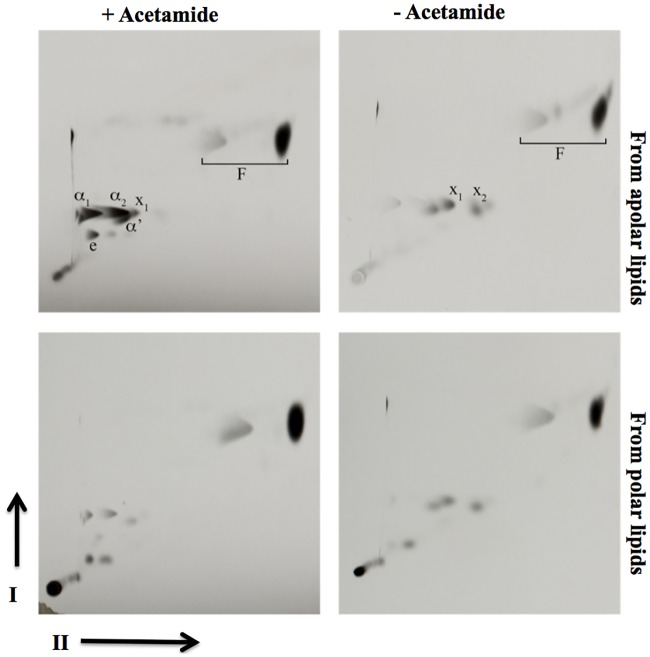
2D-argentation TLC of fatty acyl methyl esters extracted from polar and apolar lipid extracts of the Δ*MsdesA1* mutant grown in the presence or absence of acetamide. (A) Direction-I separated in petroleum ether:acetone (95:5, v/v); direction-II separated thrice in AgNO_3_ treated silica gel in petroleum ether:ethyl acetate (9:1, v/v). Mycolate species from apolar extracts are indicated and are also present in lesser amounts in the polar extracts. F; fatty acyl methyl esters.

**Fig 8:**
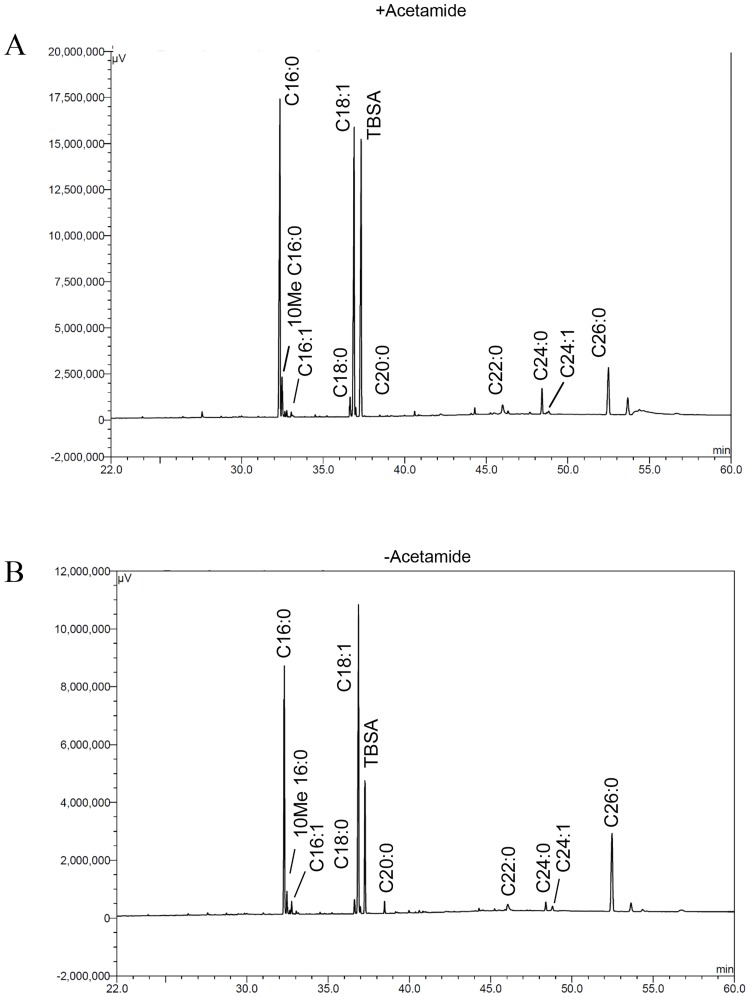
GC-MS analysis of fatty acyl methyl esters prepared from lipid extracts of the Δ*MsdesA1* mutant grown in the presence or absence of acetamide. Sigma-Aldrich fatty acyl methyl esters were used as standards. TBSA; tuberculostearic acid.

In parallel, we also analysed fatty acid methyl esters (FAMES) obtained from polar and apolar lipid extracts by 2D-argentation TLC and GC-MS. Unlike MAMEs, no additional FAME species were detected in the second dimension of the TLCs (Fig 7). We further analysed FAMEs using Gas Chromatography-Mass Spectroscopy (GC-MS). The GC-MS spectra did not reveal any major differences in unsaturated species (Fig 8), indicating that the role of *desA1* was associated with mycolic acids, but not other fatty acids found in *M*. *smegmatis*.

### Lipid analysis of the conditional mutant

**Fig 9:**
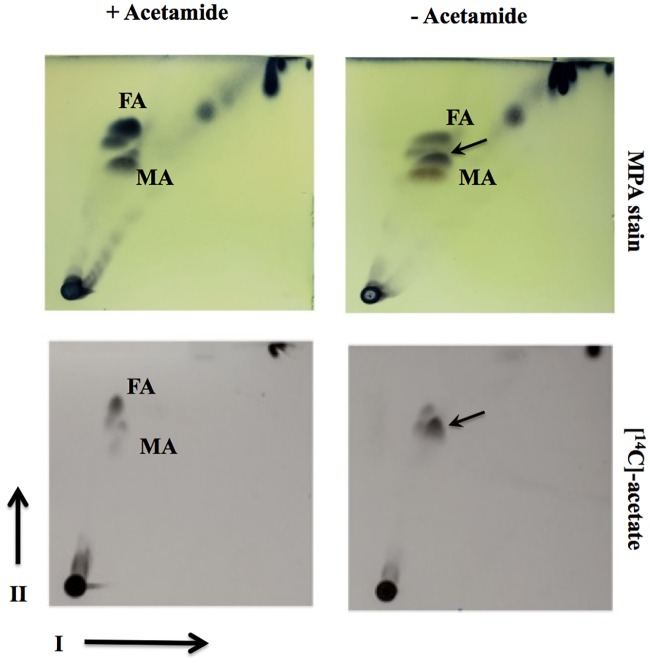
TLC analysis of apolar lipids extracted from the Δ*MsdesA1* mutant grown in the presence or absence of acetamide and separated by 2D-TLC using solvent system C. Direction-I, chloroform:methanol, 96:4 (v/v); direction-II, toluene:acetone, 80:20 (v/v). MPA, 5% ethanolic molybdophosphoric acid, followed by charring; MA, free mycolic acids; FA, free fatty acids.

**Fig 10:**
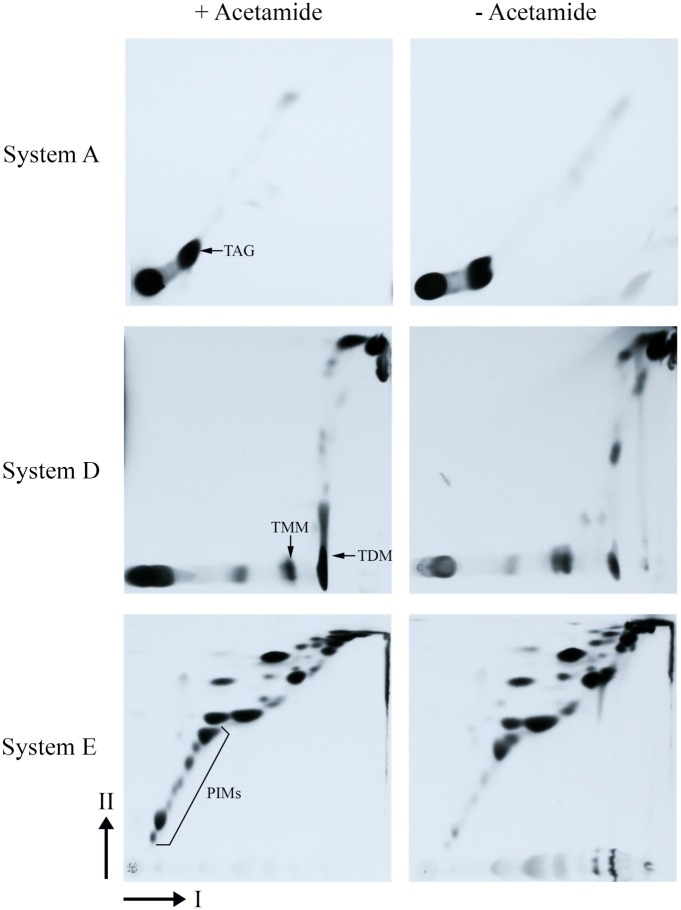
TLC analysis of apolar (Systems A and D) and polar lipids (System E) extracted from the Δ*MsdesA1* mutant grown in the presence or absence of acetamide and separated by 2D-TLC using solvent system C. System A direction I; petroleum ether:ethyl acetate 98:2 (X3), direction II; petroleum ether:acetone 98:2. System D direction I; chloroform:methanol:water 100:14:0.8, direction II; chloroform:acetone:methanol:water 50:60:2.5:3. System E direction I; chloroform:methanol:water 60:30:6, direction II; chloroform:acetic acid:methanol:water 40:25:3:6. TAG, triacylated glycerol; TMM, trehalose monoycolcate; TDM, trehalose dimycolate; PIMs, phosphatidyl inositol mannosides.

In addition to studying mycolic acid profiles of the conditional Δ*MsdesA1* mutant, we also compared the total lipid profile of the conditional mutant, grown in the presence and absence of acetamide, by 2D-TLC particularly using System C that allows visualisation of free fatty acid and free mycolic acid species [25]. While cultures of Δ*MsdesA1* grown in the presence of acetamide showed the patterns expected of the wild type strain (*M*. *smegmatis* mc^2^155), i.e. a spot each corresponding to free fatty acids (FA) and free mycolic acids (MA; Fig 9), cultures of the strain grown for 8h in the absence of acetamide showed the accumulation of a lipid species that migrated close to the free MA and FA spots (Fig 9). We then monitored the incorporation of ^14^C acetate by cultures of the conditional mutant at different time intervals in the presence or absence of acetamide. Analysis of ^14^C-labelled lipids by 2D-TLC using System C showed an accumulation of the same, unknown lipid species in cultures grown for 8h in the absence of acetamide (Fig 9). The presence of this lipid spot predominantly in the cultures growing in the absence of acetamide implied that the accumulation of the spot was directly or indirectly related to the loss of *desA1* function. Furthermore, the accumulation of this lipid species was accompanied by a decrease in the levels of free mycolic acids (Fig 9) suggesting that the accumulating lipid was related to mycolic acids. As expected, we also saw a decrease in levels of TDM after conditional depletion of DesA1 (Fig 10), but no immediate reduction was seen in other apolar lipids (Fig 10). Surprisingly, 2D-TLC of polar lipid fractions revealed relatively lower levels of PIMs in the DesA1-depleted cultures, though levels of other polar lipids remained similar.

### Loss of DesA1 leads to the accumulation of a series of fatty acids

**Fig 11:**
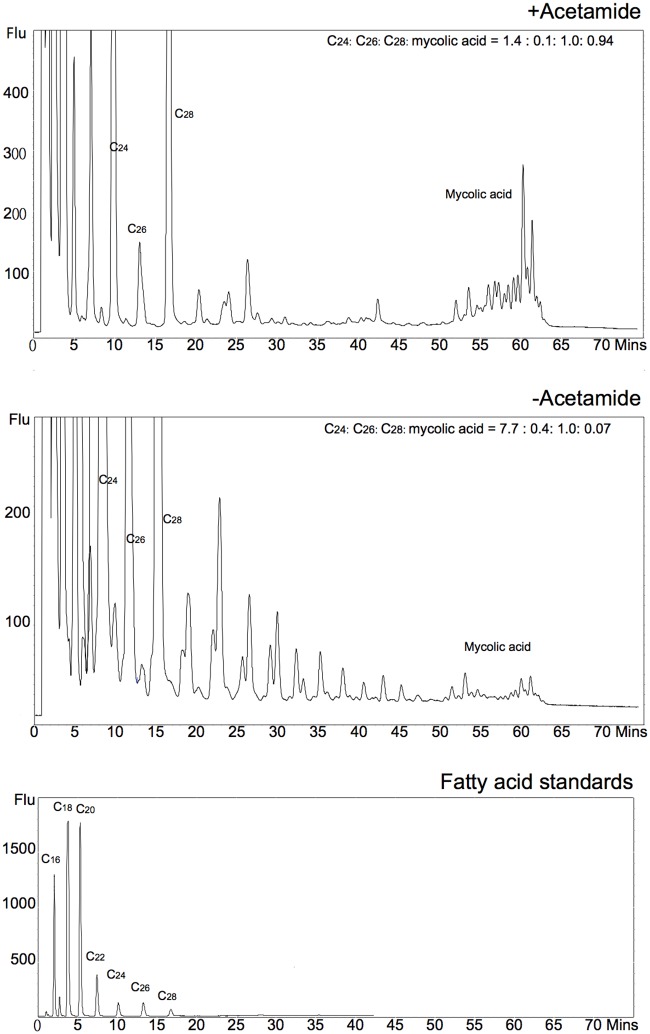
Reverse phase HPLC analysis of dimethoxycoumarin esters of fatty and mycolic acid species. Ratio of injected C_26_ internal standard to key fatty acyl species and mycolates is indicated in the inset.

The 2D-TLC patterns obtained with the Δ*MsdesA1* mutant in Fig 9 suggested that the lipid species accumulating in DesA1-depleted cells could be a fatty acyl species. To further probe the chemical composition of this lipid species, apolar free fatty acyl species (free mycolic acids, free fatty acids and unknown accumulating species) from cultures grown in the presence or absence of acetamide were extracted from preparative TLC plates (separated in System C), and converted into fluorescent dimethoxycoumarin (DMC) esters of fatty acids. The DMC esters were then analysed by reverse phase HPLC analysis (Fig 11). Differences were observed in the HPLC profiles from the extracts of cultures grown in the presence or absence of acetamide. The DesA1-depleted cultures showed the presence of a series of fatty acyl species which eluted with a retention times ranging from 15 to 50 min. Comparison of elution times to fatty acid standards and those of mycolic acids suggested that these potential fatty acyl species had chain lengths ranging from approximately C_26_ to C_48_. Thus, the apolar lipid species migrating close to the free MAs in System C consisted of a mix of fatty acid species ranging from ~C_26_-C_48_.

### The *M*. *tuberculosis desA1* ortholog is functional in *M*. *smegmatis*

**Fig 12:**
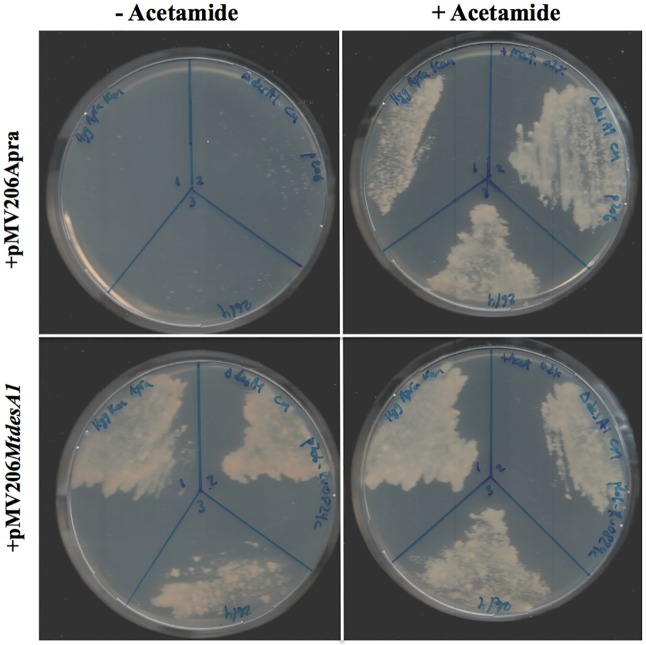
*M*. *tuberculosis desA1* rescues the growth of Δ*MsdesA1* in medium lacking acetamide. Δ*MsdesA1*strains containing either empty vector (pMV206Apra) or pMV206*MtdesA1* grown on TSB-agar plates with or without acetamide.

To test whether *M*. *tuberculosis desA1* (*Rv0824c*), could function compensate for the loss of *M*. *smegmatis desA1* in the conditional mutant, we first transformed Δ*MsdesA1* with a replicating plasmid, pMV206*MtdesA1*, containing *Rv0824c* and its native promoter. We then tested the ability of this strain to survive following growth in the absence of acetamide. Three independently obtained transformants were able to survive in the absence of acetamide in the media (Fig 12). In contrast, control strains of Δ*MsdesA1* containing only the plasmid vector were unable to grown on plates without acetamide (Fig 12). These results demonstrated that *M*. *tuberculosis desA1* was able to compensate for the loss of *M*. *smegmatis desA1* and rescue the viability of cultures of Δ*MsdesA1* grown in the absence of inducer.

## Discussion

The aim of this study was to determine whether *desA1*, one of three mycobacterial genes encoding putative aerobic desaturases, played a role in the biosynthesis of mycolic acids. We demonstrated that *MSMEG5773* (*desA1*), homologue of *M*. *tuberculosis desA1*, is an essential gene in *M*. *smegmatis*, required for growth in laboratory media. We also showed that depletion of DesA1 in a conditional mutant caused loss of mycolic acid biosynthesis and cell viability, and resulted in distinct phenotypes. The first was the accumulation of a species that co-migrated with α-MAMEs in the first dimension of 2D-TLC plates. A comparative Mass Spectroscopic analysis showed that while extracts obtained from cultures grown in the presence of absence of acetamide both contained both monoenoic and dienoic α-mycolate species (based on expected masses), depletion of DesA1 led to change in the relative proportion of the two classes, with levels of the potential monoenoic forms increasing. A conclusive role in double bond addition, and potential identification of double bond position would require a fine structural analysis of these unique species using NMR, however bacterial cell lysis in DesA1-depleted cultures limited our ability to conduct these studies, which would require milligram quantities of purified lipid.

In addition to the phenotype described above, we also observed fatty acyl species of sizes ranging from ~C_28_-C_48_ in apolar lipid extracts of cells of the conditional mutant following depletion of DesA1, with a concurrent reduction in levels of free mycolic acids. As FAS-I derived fatty acids are not known to extend beyond C_26_, these intermediate length fatty acyl species (~C_28_-C_48_) were thus likely to be truncated mero-mycolate species produced by aborted FAS-II elongation events resulting from a loss of DesA1-catalysed desaturation. Thus, while monenoic α-mycolate-related species were initially identified in DesA1 depleted cultures, it was likely that FAS-II mediated merochain elongation was not efficient in the absence of DesA1 and led to the observed series of truncated fatty acyl derivatives. These findings co-relate with earlier hypothesis that desaturation of mycolic acids occurs during merochain elongation [3,26].

We also observed two unexpected phenotypes in the conditional mutant. The first was that levels of a cyclopropanated derivative of α-mycolates remained unaffected by the depletion of DesA1. This was an unexpected outcome as double bond formation precedes cyclopropanation. Fatty acid cyclopropanation is known to increase cell envelope rigidity and it is likely that additional cyclopropanation occurs in residual α-mycolates in lysing cultures to counteract damage to the cell envelope following overall depletion of mycolic acid biosynthesis. The second unexpected outcome of DesA1 depletion was the reduction in the levels of PIMs (Fig 10). This was surprising, as we did not see significant alterations in levels of fatty acids that comprise PIMs in the GC-MS analysis (Fig 8). It seems likely that the observed effect of DesA1 depletion on PIMs is an indirect one.

Our studies have identified *desA1* as a gene that plays a role in mycolic acid biosynthesis. Also, the conditional loss of DesA1 eventually led to cell death, and the viability of the strain could be rescued upon introduction of plasmid-borne *M*. *tuberculosis desA1*, highlighting the potential of DesA1 as a new anti-*M*. *tuberculosis* drug target.

## Materials and Methods

### Generation and characterisation of a *M*. *smegmatis MSMEG5773* (*desA1*) conditional mutant

The genetic tool CESTET was used to generate a *M*. *smegmatis* conditional Δ*MsdesA1* mutant [27]. *MSMEG5773* was PCR amplified from *M*. *smegmatis* mc^2^155 genomic DNA and cloned downstream of an acetamidase promoter fragment into the integrating vector pMV306 [28], to generate pMV306-*MSMEG5773*. *M*. *smegmatis* mc^2^155 [29] was electroporated with pMV306-*MSMEG5773* to generate a merodiploid strain mc^2^155::pMV306-*MSMEG5773*. In parallel, phΔ*MSMEG5773*, a temperature sensitive, recombinant phage designed to replace *MSMEG5773* with a hygromycin resistance marker (*hyg*), was generated using protocols described by Larsen *et al*. [20]. Specialized Transduction using the merodiploid strain and phΔ*MSMEG56773* was done as previously described [21,27]. Transductants were selected at the non-permissive temperature of 37°C on selective plates containing 150 μg/ml hygromycin and 0.02% acetamide. Replacement of the native copy of *MSMEG5773* by *hyg* was confirmed by Southern blot. One bonafide knockout, designated as Δ*MsdesA1*, was selected for further analysis. Conditional depletion of MSMEG5773 (DesA1) in Δ*MsdesA1* to monitor mycolic acids and other lipids was done using methods described previously [27].

### Complementation of Δ*MsdesA1* with *M*. *tuberculosis desA1*

*M*. *tuberculosis desA1* (*Rv0824c*) was PCR amplified along with promoter sequences 300bp upstream of the start codon, from genomic *M*. *tuberculosis* H37Rv genomic DNA and cloned into pMV206Apra, an *E*. *coli-Mycobacterium* shuttle plasmid [30]. The plasmid was introduced into Δ*MsdesA1* by electroporation [30]. Three independent apramycin resistant transformants were tested for their ability to grow on TSB-agar plates, with or without acetamide. The Δ*MsdesA1* strain was also transformed with pMV206Apra for use as a control strain.

### Extraction and characterisation of mycolic acids and other lipids

Extraction of polar, apolar lipids and mycolic acid methyl esters (MAMEs) from the conditional mutant, and subsequent TLC analysis was carried out using protocols described by Dobson *et al*. [25]. Dry weights of cell pellets were used as a measure to equalise loading on TLC plates. Argentation TLC was carried out as described earlier [28]. Characterisation of the unknown lipid observed in Fig 9 was done by preparative TLC of long-chain acidic components in the apolar lipid using toluene/acetone, 8:2. To convert the acidic components into fluorescent dimethoxycoumarin (DMC) esters [31], a 0.4% solution of 4-bromomethyl-6,7-dimethoxycoumarin in dichloromethane, was allowed to react with the extracts overnight under phase-transfer conditions [25,32]. 0.1mg of each sample was dissolved in 50μl heptane. 20μl of each sample was placed in a new vial. A concentration of 40μg/1000μl DMC derivatised C28 was used as an internal control. 20μl of the internal control was added to vial prior to HPLC analysis. DMC-esters of saturated C_20_, C_26_ and C_28_ fatty acids were used as standards. DMC esters were separated on an Alltech 205250 C_18_ reverse phase cartridge, using a water-methanol/dichloromethane elution sequence. Purified DMC esters were eluted by 100% DCM and analysed by reverse phase high performance liquid chromatography (HPLC). HPLC analyses used a VWR Hitachi Elite Pump L-2130, LaChrom Autosampler L-2200, Column Oven L-2300, Fluorescence Detector L-2480 and Foxy Jr. Fraction Collector; data were processed by Scientific Software Inc. EZChrom Elite, version 3.1.6 software. A reverse phase (Alltech 81412 Alltima C18, 3μm 50 x 4.6mm) column was used. HPLC analysis conditions are shown below. Samples were dissolved in HPLC grade heptane (500μl) and 1 to 5μl was injected. Using a flow rate of 1ml/min, a gradient of acetonitrile/tetrahydrofuran; 100:0 to 40:60 over 70 min was used for elution. The ratio of DMC derivatised C24, 26, 28 and mycolic acids (MA) were calculated by the area under peaks from the HPLC profiles. The ratio of the net internal standard C28: MA were calculated by subtracting the background C28 from the total C28.

The GC-FID analysis of fatty acid methyl esters (FAMEs) was performed on a Shimadzu GC-2010. The FAMEs separation was achieved using a DB-225 column (30 m x 0.25 mm i.d., 0.25 μm film thickness, J&W Scientific) with a column flow of 1.26ml/min (Helium at a constant pressure of 100kPa). Injector and detector temperatures were 50°C. The Injection volume was 5ul. The column temperature was programmed initially at 50°C for 2 mins, increased at the rate of 4°C/min to 220°C, and then maintained at 220°C for a further 30.5 mins. The total program time was 75 mins.

### Purification and mass analysis of MAMEs

MAMEs were detected on TLC described previously [33]. The TLC was developed with the solvent system; benzene or *n*-hexane/diethylether (90:15, vol/vol), three runs. The MAMEs were purified from the TLC plate. To assign the structures, the molecular weight of each MAME was measured by MALDI Spiral-TOF neIons generated by irradiation with a 349-nm Nd:YLF laser were accelerated at 20 kV. The settings for delay time and grid voltage were optimized to stay constant at *ΔM*<*ca*. 0.03 Da at full width at half maximum (FWHM) over the range of *m*/*z* 500 to 1500. The matrix was 10 mg/mL 2,5-dihydroxybenzoic acid in tetrahydrofuran (THF). A sodium iodide solution of 1 mg/mL in THF was used as a cationization agent. MALDI mass spectra were observed in positive spiral mode by averaging 1500 individual laser shots. Three or five mass spectra for each sample were collected. The structures of MAMEs were assigned by the Rf value of TLC and the molecular weight.

## Supporting Information

S1 Fig Spiral MALDI-TOF of MAMES isolated from Δ*MsdesA1*. (PDF)

S2 Fig Spiral MALDI-TOF of MAMES isolated from Δ*MsdesA1*. (PDF)

S1 Table Spiral MALDI-TOF of MAMES isolated from Δ*MsdesA1*. (PDF)

S2 Table Spiral MALDI-TOF of MAMES isolated from Δ*MsdesA1*. (PDF)

## References

[1] Marrakchi H, Laneelle MA, Daffe M (2014) Mycolic acids: structures, biosynthesis, and beyond. Chem Biol 21: 67–85. 10.1016/j.chembiol.2013.11.011 24374164

[2] Nataraj V, Varela C, Javid A, Singh A, Besra GS, et al (2015) Mycolic acids: deciphering and targeting the Achilles' heel of the tubercle bacillus. Mol Microbiol. 10.1111/mmi.13101 26135034 PMC4949712

[3] Takayama K, Wang C, Besra GS (2005) Pathway to synthesis and processing of mycolic acids in *Mycobacterium tuberculosis*. Clin Microbiol Rev 18: 81–101. 10.1128/CMR.18.1.81-101.2005 15653820 PMC544180

[4] Gande R, Gibson KJ, Brown AK, Krumbach K, Dover LG, et al (2004) Acyl-CoA carboxylases (*accD2* and *accD3*), together with a unique polyketide synthase (*Cg-pks*), are key to mycolic acid biosynthesis in Corynebacterianeae such as *Corynebacterium glutamicum* and *Mycobacterium tuberculosis*. J Biol Chem 279: 44847–44857. 10.1074/jbc.M408648200 15308633

[5] Portevin D, De Sousa-D'Auria C, Houssin C, Grimaldi C, Chami M, et al (2004) A polyketide synthase catalyzes the last condensation step of mycolic acid biosynthesis in mycobacteria and related organisms. Proc Natl Acad Sci U S A 101: 314–319. 10.1073/pnas.0305439101 14695899 PMC314182

[6] Bhatt A, Brown AK, Singh A, Minnikin DE, Besra GS (2008) Loss of a mycobacterial gene encoding a reductase leads to an altered cell wall containing beta-oxo-mycolic acid analogs and accumulation of ketones. Chem Biol 15: 930–939. 10.1016/j.chembiol.2008.07.007 18804030 PMC2568869

[7] Lea-Smith DJ, Pyke JS, Tull D, McConville MJ, Coppel RL, et al (2007) The reductase that catalyzes mycolic motif synthesis is required for efficient attachment of mycolic acids to arabinogalactan. J Biol Chem 282: 11000–11008. 10.1074/jbc.M608686200 17308303

[8] Dubnau E, Laneelle MA, Soares S, Benichou A, Vaz T, et al (1997) *Mycobacterium bovis* BCG genes involved in the biosynthesis of cyclopropyl keto- and hydroxy-mycolic acids. Mol Microbiol 23: 313–322. 9044265 10.1046/j.1365-2958.1997.2301589.x

[9] Glickman MS, Cahill SM, Jacobs WR Jr. (2001) The *Mycobacterium tuberculosis cmaA2* gene encodes a mycolic acid trans-cyclopropane synthetase. J Biol Chem 276: 2228–2233. 10.1074/jbc.C000652200 11092877

[10] Glickman MS, Cox JS, Jacobs WR Jr. (2000) A novel mycolic acid cyclopropane synthetase is required for cording, persistence, and virulence of *Mycobacterium tuberculosis*. Mol Cell 5: 717–727. 10.1016/S1097-2765(00)80250-6 10882107

[11] Asselineau CP, Lacave CS, Montrozier HL, Prome JC (1970) Structural relations between unsaturated mycolic acids and short-chain unsaturated acids synthesized by *Mycobacterium phlei*: Metabolic implications. Eur J Biochem 14: 406–410. 5479374 10.1111/j.1432-1033.1970.tb00304.x

[12] Mohan S, Kelly TM, Eveland SS, Raetz CR, Anderson MS (1994) An *Escherichia coli* gene (FabZ) encoding (3R)-hydroxymyristoyl acyl carrier protein dehydrase. Relation to fabA and suppression of mutations in lipid A biosynthesis. J Biol Chem 269: 32896–32903. 7806516

[13] Zhang YM, Marrakchi H, White SW, Rock CO (2003) The application of computational methods to explore the diversity and structure of bacterial fatty acid synthase. J Lipid Res 44: 1–10. 10.1194/jlr.R200016-JLR200 12518017

[14] Cole ST, Brosch R, Parkhill J, Garnier T, Churcher C, et al (1998) Deciphering the biology of *Mycobacterium tuberculosis* from the complete genome sequence. Nature 393: 537–544. 10.1038/31159 9634230

[15] Phetsuksiri B, Jackson M, Scherman H, McNeil M, Besra GS, et al (2003) Unique mechanism of action of the thiourea drug isoxyl on *Mycobacterium tuberculosis*. J Biol Chem 278: 53123–53130. 10.1074/jbc.M311209200 14559907 PMC4747054

[16] Cole ST, Eiglmeier K, Parkhill J, James KD, Thomson NR, et al (2001) Massive gene decay in the leprosy bacillus. Nature 409: 1007–1011. 10.1038/35059006 11234002

[17] Garnier T, Eiglmeier K, Camus JC, Medina N, Mansoor H, et al (2003) The complete genome sequence of *Mycobacterium bovis*. Proc Natl Acad Sci U S A 100: 7877–7882. 10.1073/pnas.1130426100 12788972 PMC164681

[18] Dyer DH, Lyle KS, Rayment I, Fox BG (2005) X-ray structure of putative acyl-ACP desaturase DesA2 from *Mycobacterium tuberculosis* H37Rv. Protein Sci 14: 1508–1517. 10.1110/ps.041288005 15929999 PMC2253383

[19] Bardarov S, Bardarov S Jr, Pavelka MS Jr, Sambandamurthy V, Larsen M, et al (2002) Specialized transduction: an efficient method for generating marked and unmarked targeted gene disruptions in *Mycobacterium tuberculosis*, *M*. *bovis* BCG and *M*. *smegmatis*. Microbiology 148: 3007–3017. 10.1099/00221287-148-10-3007 12368434

[20] Larsen MH, Biermann K, Tandberg S, Hsu T, Jacobs WR Jr. (2007) Genetic Manipulation of *Mycobacterium tuberculosis*. Curr Protoc Microbiol Chapter 10: Unit 10A 12 10.1002/9780471729259.mc10a02s6 18770603

[21] Bhatt A, Jacobs WR Jr. (2009) Gene essentiality testing in *Mycobacterium smegmatis* using specialized transduction. Methods Mol Biol 465: 325–336. 10.1007/978-1-59745-207-6_22 20560066

[22] Slayden RA, Lee RE, Armour JW, Cooper AM, Orme IM, Brennan PJ, Besra GS. (1996) Antimycobacterial action of thiolactomycin: an inhibitor of fatty acid and mycolic acid synthesis. Antimicrob Agents Chemother 40:2813–2819. 9124847 10.1128/aac.40.12.2813 PMC163628

[23] Teramoto K, Suga M., Sato T., Wada T., Yamamoto A. and Fujiwara N. (2015) Characterization of Mycolic Acids in Total Fatty Acid Methyl Ester Fractions from *Mycobacterium* Species by High Resolution MALDI-TOFMS. Mass Spectrometry 4 10.5702/massspectrometry.A0035 26819906 PMC4541030

[24] Teramoto K, Tamura T, Hanada S, Sato T, Kawasaki H, et al (2013) Simple and rapid characterization of mycolic acids from Dietzia strains by using MALDI spiral-TOFMS with ultra high mass-resolving power. J Antibiot (Tokyo) 66: 713–717. 10.1038/ja.2013.79 23981960

[25] Dobson G, Minnikin D.E., Minnikin S.M., Parlett M., Goodfellow M., Ridell M. et al (1985) Systematic analysis of complex mycobacterial lipids Chemical Methods in Bacterial Systematics. London: Academic Press pp. 237–265.

[26] Watanabe M, Aoyagi Y, Mitome H, Fujita T, Naoki H, et al (2002) Location of functional groups in mycobacterial meromycolate chains; the recognition of new structural principles in mycolic acids. Microbiology 148: 1881–1902. 10.1099/00221287-148-6-1881 12055308

[27] Bhatt A, Kremer L, Dai AZ, Sacchettini JC, Jacobs WR Jr. (2005) Conditional depletion of KasA, a key enzyme of mycolic acid biosynthesis, leads to mycobacterial cell lysis. J Bacteriol 187: 7596–7606. 10.1128/JB.187.22.7596-7606.2005 16267284 PMC1280301

[28] Stover CK, de la Cruz VF, Fuerst TR, Burlein JE, Benson LA, et al (1991) New use of BCG for recombinant vaccines. Nature 351: 456–460. 10.1038/351456a0 1904554

[29] Snapper SB, Melton RE, Mustafa S, Kieser T, Jacobs WR Jr. (1990) Isolation and characterization of efficient plasmid transformation mutants of *Mycobacterium smegmatis*. Mol Microbiol 4: 1911–1919. 10.1111/j.1365-2958.1990.tb02040.x 2082148

[30] Bernut A, Viljoen A, Dupont C, Sapriel G, Blaise M, Bouchier C, Brosch R, de Chastellier C, Herrmann JL, Kremer L. (2016) Insights into the smooth-to-rough transitioning in *Mycobacterium bolletii* unravels a functional Tyr residue conserved in all mycobacterial MmpL family members. Mol Microbiol 99:866–883. 10.1111/mmi.13283 26585558

[31] Hagen SR, Thompson JD (1995) Analysis of mycolic acids by high-performance liquid chromatography and fluorimetric detection. Implications for the identification of mycobacteria in clinical samples. J Chromatogr A 692: 167–172. 10.1016/0021-9673(94)00743-S 7719452

[32] Hershkovitz I, Donoghue HD, Minnikin DE, Besra GS, Lee OY, et al (2008) Detection and molecular characterization of 9,000-year-old *Mycobacterium tuberculosis* from a Neolithic settlement in the Eastern Mediterranean. PLoS One 3: e3426 10.1371/journal.pone.0003426 18923677 PMC2565837

[33] Bhatt A, Fujiwara N, Bhatt K, Gurcha SS, Kremer L, et al (2007) Deletion of kasB in *Mycobacterium tuberculosis* causes loss of acid-fastness and subclinical latent tuberculosis in immunocompetent mice. Proc Natl Acad Sci U S A 104: 5157–5162. 10.1073/pnas.0608654104 17360388 PMC1829279

