# Supplementary figures and images for: Identification of a Desaturase Involved in Mycolic Acid Biosynthesis in Mycobacterium smegmatis
Source: PLoS One. 2016 Oct 14;11(10):e0164253. doi: 10.1371/journal.pone.0164253 (PMC5065219; doi:10.1371/journal.pone.0164253)

S1 Fig. Spiral MALDI-TOF of MAMES isolated from *ΔMsdesA1*

+ Acetamide

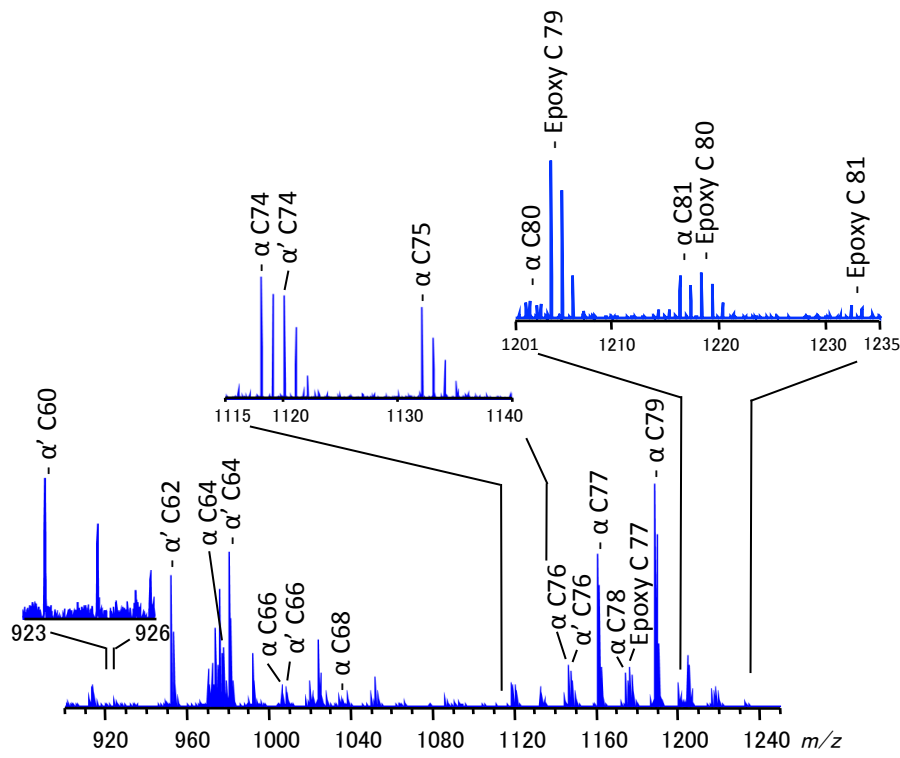

- Acetamide

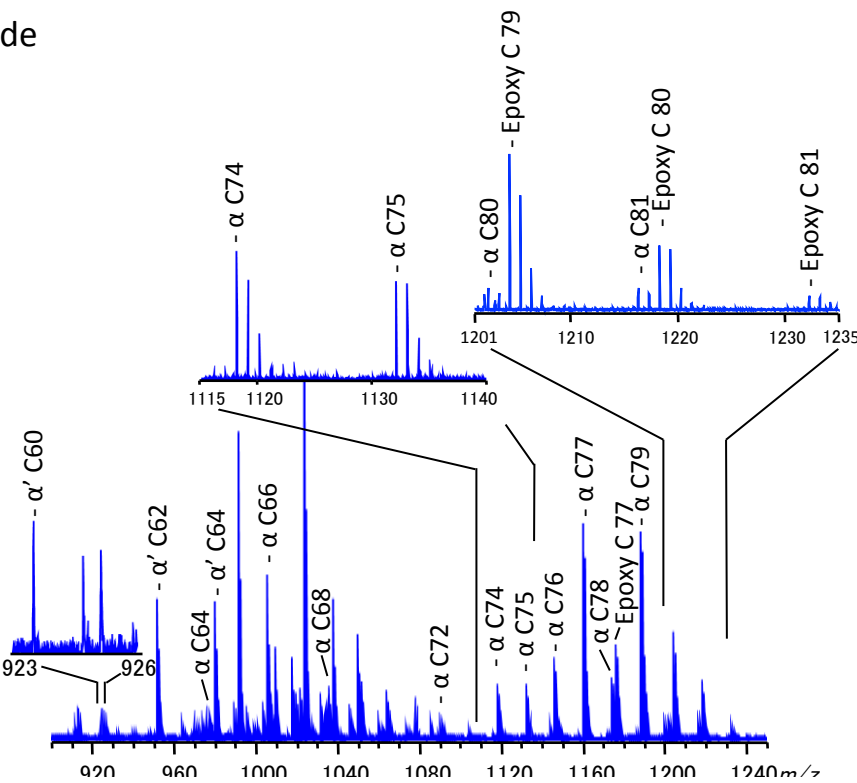

Supplement: S1 Fig — (PDF) [file pone.0164253.s001.pdf]

S2 Fig. Spiral MALDI-TOF of MAMES isolated from  $\Delta MsdesA1$

+ Acetamide

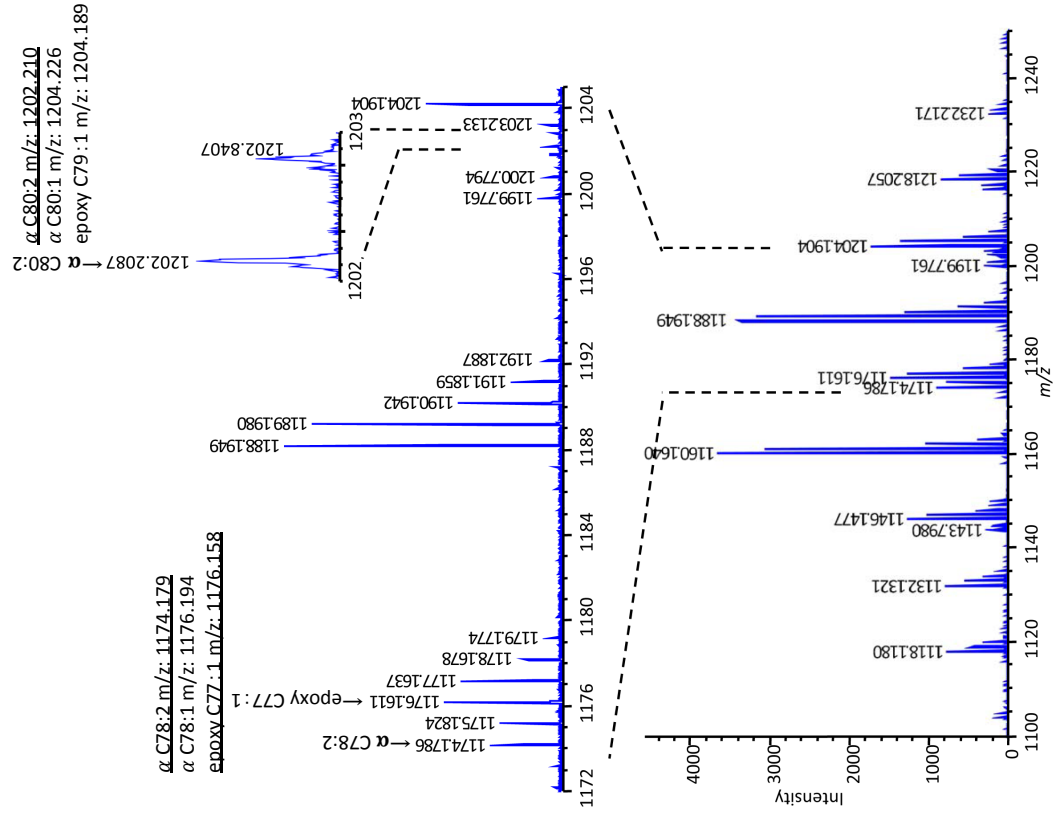

- Acetamide

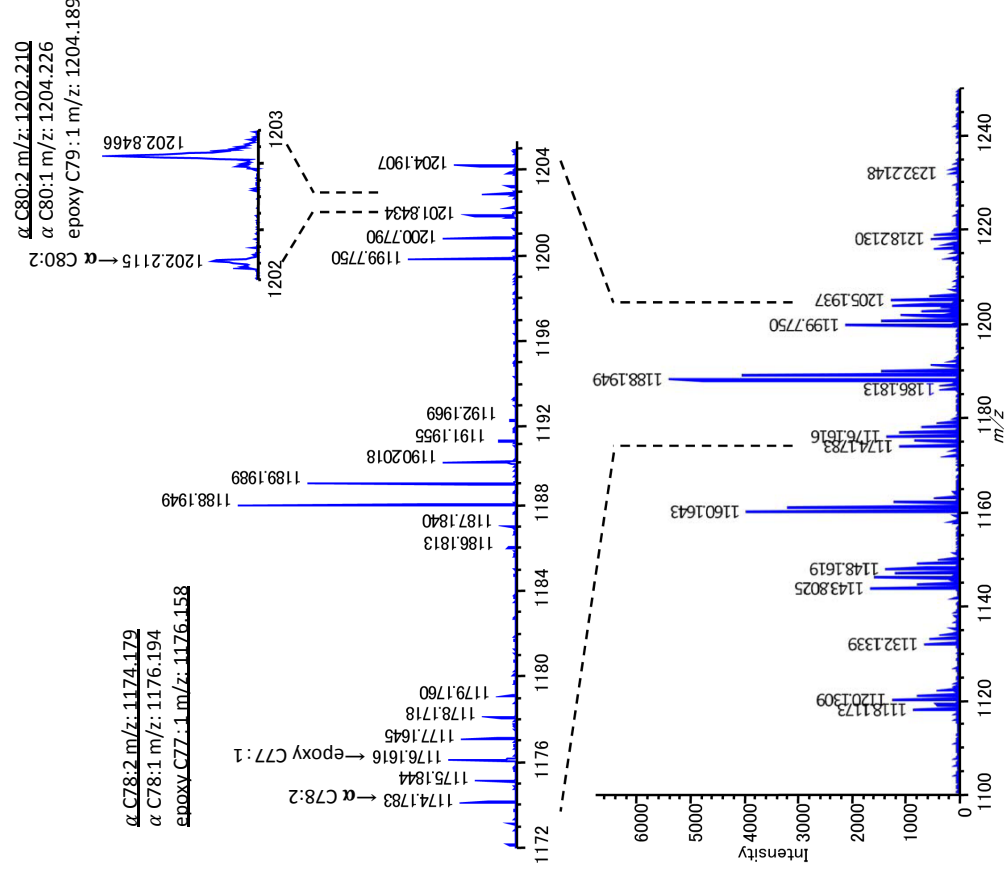

Supplement: S2 Fig — (PDF) [file pone.0164253.s002.pdf]
