# Supplementary material for: Identification of a Desaturase Involved in Mycolic Acid Biosynthesis in Mycobacterium smegmatis
Source: PLoS One. 2016 Oct 14;11(10):e0164253. doi: 10.1371/journal.pone.0164253 (PMC5065219; doi:10.1371/journal.pone.0164253)
Supplement: S1 Table — (PDF) [file pone.0164253.s003.pdf]

S1 Table. Spiral MALDI-TOF of MAMES isolated from Δ*MsdesA1*

+Acetamide

Peak ratio (maximum peak of the species, C79:2=100%)

Carbon number	α-MA		α'-MA		Epoxy-MA
	dienoic	monoenoic	monoenoic	saturated	
60	0.0	0.0	0.0	6.3	0.0
62	0.0	0.0	0.0	47.4	0.0
64	0.0	0.0	3.5	44.8	0.0
66	0.0	0.0	10.7	3.8	0.0
68	0.0	0.0	15.8	0.0	0.0
70	0.0	0.0	0.5	0.0	0.0
72	4.7	0.0	0.0	0.0	0.0
74	18.9	0.0	0.0	0.0	0.0
75	16.8	0.0	0.0	0.0	5.8
76	30.6	0.0	0.0	0.0	0.0
77	94.1	0.0	0.0	0.0	41.3
78	23.7	0.0	0.0	0.0	14.1
79	100.0	0.0	0.0	0.0	44.8
80	8.4	0.0	0.0	0.0	20.9
81	8.0	0.0	0.0	0.0	5.1

-Acetamide

Peak ratio (maximum peak of the species, C79:2=100%)

Carbon number	α-MA		α'-MA		Epoxy-MA
	dienoic	monoenoic	monoenoic	saturated	
60	0.0	0.0	0.0	2.1	0.0
62	0.0	0.0	0.3	43.3	0.0
64	0.0	0.0	5.6	52.4	0.0
66	0.0	0.0	4.8	3.6	0.0
68	0.0	0.0	4.8	0.0	0.0
70	0.0	0.0	0.0	0.0	0.0
72	0.7	1.3	0.0	0.0	0.0
74	9.1	6.1	0.0	0.0	0.0
75	6.4	0.0	0.0	0.0	0.0
76	21.1	6.5	0.0	0.0	0.0
77	65.1	0.0	0.0	0.0	21.4
78	13.3	0.0	0.0	0.0	0.0
79	100.0	0.0	0.0	0.0	23.1
80	3.7	0.0	0.0	0.0	6.7
81	5.8	0.0	0.0	0.0	2.3
