# Supplementary material for: Identification of a Desaturase Involved in Mycolic Acid Biosynthesis in Mycobacterium smegmatis
Source: PLoS One. 2016 Oct 14;11(10):e0164253. doi: 10.1371/journal.pone.0164253 (PMC5065219; doi:10.1371/journal.pone.0164253)
Supplement: S2 Table — (PDF) [file pone.0164253.s004.pdf]

S2 Table. Spiral MALDI-TOF of MAMES isolated from Δ*MsdesA1*

		-Acetamide total MAs		+Acetamide total MAs	
	m/z Cal.	*m/z* Obs.	Int.	*m/z* Obs.	Int.
α′-MA					
60	923.914	923.916	0.5	923.913	1.2
62	951.945	951.946	10.6	951.9453	9.0
64	979.976	979.976	12.8	979.9767	8.3
66	1008.007	1008.006	0.9	1008.007	0.1
72	1092.101	1092.099	0.3	-	-
74	1120.133	1120.131	1.5	-	-
76	1148.164	1148.159	1.6	-	-
α-MA					
62	949.929	949.927	0.1	-	-
64	977.960	977.962	1.4	977.9655	0.6
66	1005.992	1005.992	1.2	1005.995	2.4
68	1034.023	1034.023	1.2	1034.025	3.0
70	1062.054	-	-	1062.057	0.1
72	1090.086	1090.087	0.2	1090.087	0.6
74	1118.117	1118.118	2.2	1118.118	3.5
75	1132.133	1132.133	1.6	1132.133	3.2
76	1146.148	1146.148	5.1	1146.145	6.7
77	1160.164	1160.164	15.9	1160.164	17.6
78	1174.180	1174.175	3.2	1174.168	3.0
79	1188.195	1188.194	24.4	1188.191	21.5
80	1202.211	1202.206	0.9	1202.198	1.1
81	1216.227	1216.222	1.4	1216.213	1.1
Epoxy-MA					
77	1176.159	1176.164	5.2	1176.162	5.8
79	1204.190	1204.192	5.7	1204.191	7.4
80	1218.206	1218.209	1.6	1218.206	3.2
81	1232.221	1232.220	0.6	1232.217	0.8
